# DNet: Dynamic Neighborhood Feature Learning in Point Cloud

**DOI:** 10.3390/s21072327

**Published:** 2021-03-26

**Authors:** Fujing Tian, Zhidi Jiang, Gangyi Jiang

**Affiliations:** Faculty of Information Science and Engineering, Ningbo University, Ningbo 315211, China; tianfujing@foxmail.com (F.T.); jiangzhidi@nbu.edu.cn (Z.J.)

**Keywords:** point cloud, dynamic neighborhood, feature learning, attention mechanism, masking mechanism

## Abstract

Neighborhood selection is very important for local region feature learning in point cloud learning networks. Different neighborhood selection schemes may lead to quite different results for point cloud processing tasks. The existing point cloud learning networks mainly adopt the approach of customizing the neighborhood, without considering whether the selected neighborhood is reasonable or not. To solve this problem, this paper proposes a new point cloud learning network, denoted as Dynamic neighborhood Network (DNet), to dynamically select the neighborhood and learn the features of each point. The proposed DNet has a multi-head structure which has two important modules: the Feature Enhancement Layer (FELayer) and the masking mechanism. The FELayer enhances the manifold features of the point cloud, while the masking mechanism is used to remove the neighborhood points with low contribution. The DNet can learn the manifold features and spatial geometric features of point cloud, and obtain the relationship between each point and its effective neighborhood points through the masking mechanism, so that the dynamic neighborhood features of each point can be obtained. Experimental results on three public datasets demonstrate that compared with the state-of-the-art learning networks, the proposed DNet shows better superiority and competitiveness in point cloud processing task.

## 1. Introduction

With the rapid development of three dimensional (3D) sensing technologies, using deep learning to understand and analyze point clouds is becoming one of the important research topics [1,2,3]. As the output of 3D sensor, point cloud is composed of much number of points in 3D space. The neighborhood of point cloud is similar to the neighborhood of pixels in image, but point cloud does not have the regular grid structure as the image [4,5]. For learning-based point cloud processing, too large a neighborhood may lead to incorrect learning, but too small a neighborhood cannot ensure sufficient information being included for learning.

In recent years, deep learning has made great progress in point cloud classification and segmentation [6,7], and the existing methods can be roughly divided into the multi-view approach, the voxel approach, the graph convolution approach, and the point set approach. The multi-view approach projects point cloud to 2D plane from multiple angles to generate image data, then the traditional Convolutional Neural Network (CNN) is used for feature learning [8,9,10]. For this kind of approach, when the objects in the scene are obscured or the point density changes, the accuracy of object classification and segmentation will be reduced. The voxel approach converts point cloud into regular 3D meshes, and then processes the meshes with 3D convolutions [11,12]. However, the voxel approach is greatly limited because of the reduced resolution resulted from quantization, a large amount of data preprocessing and the computational complexity of 3D convolution. In addition, the voxels of point cloud will make 3D convolution away from the surface of the point cloud, leading to the loss of effective surface information. Riegler et al. [13] and Klokov et al. [14] used different spatial segmentation methods to solve the problem of computational complexity. However, these methods still rely on the accuracy of spatial segmentation and cannot effectively extract surface features of point cloud, thus they may lose some information of the fine-grained geometric manifolds.

Since the points in point cloud are similar to the nodes in graph, some works used graph convolution approaches to process the point cloud [15,16,17,18,19]. The graph convolution approach can be divided into the spectral convolution and the spatial convolution [7]. The spectral convolution method uses the eigenvector decomposition of the Laplacian matrix, and then obtains the global descriptor of the point cloud through network learning, based on which the classification and segmentation of the point cloud can be achieved [15]. Since the Laplacian matrix of each point cloud should be calculated, the computational cost is huge. At the same time, because the spectral convolution is associated with the Laplacian matrix, its generalization ability is weak. By contrast, the spatial convolution approach can directly perform convolution on the local neighborhoods of point cloud [16,17,18,19], and has high computational efficiency and strong generalization.

The point set approach can learn features from point cloud directly and efficiently. Qi et al. [20] designed the PointNet, which learns each point individually and uses a symmetric max-pooling function to maintain the permutation invariance of the points. In the PointNet, the network only considers every point itself, without combining its neighborhood information. To improve the PointNet, Qi et al. [21] further proposed the PointNet++ with a multi-scale mechanism to capture multi-scales local regions. Graham et al. [22] constrained the execution of volumetric convolution only along the input sparse set of active voxels of the grid. Hua et al. [23] put the points into a kernel unit, and then convolved the point cloud with the kernel weights. Su et al. [24] mapped the input data to a high-dimensional grid and processed it using bilateral convolution. Li et al. [25] proposed learning the X-transform from the input point cloud, and then obtained the invariant feature of point cloud permutation with traditional convolution. Huang et al. [26] designed the RSNet, which projects unordered points onto an ordered sequence of feature vectors through a slice pooling layer, and then used Recurrent Neural Network (RNN) to learn the sequence. Tchapmi et al. [27] combined trilinear interpolation and conditional random fields to perform segmentation on point clouds. Li et al. [28] simulated the spatial distribution of point cloud by establishing a self-organizing map (SOM), and then extracted the hierarchical features from SOM nodes. Huang et al. [29] used multi-scale point embedding, manifold learning and global graph-based optimization to deal with laser scanning point clouds.

For the point set approach, in order to learn the features of point cloud more effectively, many methods have been proposed. Wu et al. [6] regarded the convolution kernel as a non-linear function of local coordinates composed of a weight function and a density function, and then used it to convolve point cloud. Xu et al. [7] processed irregular data through the parameterized filters. Groh et al. [30] extended the traditional convolution to larger scale point cloud processing through exploring different parameterizations to generate the edge-dependent filters. Verma et al. [31] used soft-assignment matrices to extend traditional convolution into point cloud. Wang et al. [32] proposed a learnable operator to learn feature from non-grid structured data. Hermosilla et al. [33] proposed the density-based 3D convolution Markov approximation, which is used to learn the features of non-uniform point clouds. Shen et al. [34] defined the point set kernel as a set of learnable 3D points by measuring the geometric relationship between adjacent points, and then used the point set kernel to extract the feature of point cloud.

Although the methods mentioned above can be used to learn point clouds, most of them have the problem that feature extraction of local regions is rough because only simple regular range (such as the *k*-nearest neighborhood, spherical neighborhood, etc.) is defined as the neighborhood, without considering the semantics of the neighborhood. To solve this problem, this paper proposes a Dynamic neighborhood Network (DNet) with an adaptive selection strategy of the neighborhood. Firstly, the single-head structure is designed to obtain the attention weight of the neighborhood by learning the self-features, manifold features and neighborhood features of the point cloud. Then, the mask mechanism is used to remove some pseudo neighborhood points, and the dynamic neighborhood features are obtained. Finally, the multi-head structure is utilized to learn features in different neighborhood range so that multi-scale features can be obtained. The contributions of this paper are as follows:

- To learn the features of different scales of a point cloud, a multi-head structure is designed to effectively capture multi-scale features, and the Feature Enhancement Layer (FELayer) inside each head supplements the manifold features of local regions of the point cloud, so that each head can learn enough contextual information;
- An attention mechanism is proposed to obtain the contribution degree of each neighborhood point in a local region through learning the self-features, 2D manifold features and neighborhood features of the local region;
- A masking mechanism is designed to remove the pseudo neighborhood points that may mislead the neighborhood learning but keep the ones which are conducive to network understanding, so that the network can learn neighborhood features more reasonably and effectively.

The rest of this paper is organized as follows. Section 2 analyzes the motivation of this paper, and the proposed method is described in detail in Section 3. Section 4 gives the comparison results of the DNet and the state-of-the-art point cloud classification and segmentation networks. Section 5 concludes this paper.

## 2. Motivation

In this section, the works of point cloud neighborhood learning are reviewed. Then, the difference between the proposed attention mechanism and some traditional attention networks is introduced. Finally, the neighborhood problem worth thinking about and the motivation of this paper are put forward.

Local feature of point cloud is very important to understanding point cloud. For determining the neighborhood of a point in point cloud, most existing methods usually calculate the *k*-Nearest Neighbor (*k*-NN) points or use the spherical neighborhood with radius *r*, and then learn features on the neighborhood. For the neighborhood learning, PointNet++ [21] divided point cloud into multiple spherical neighborhoods to extract multi-scale context information. Wang et al. [35] proposed a dynamic graph CNN (DGCNN) to aggregate the features learned from local regions by calculating the *k*-NN points of each point. Thomas et al. [36] defined a new multi-scale neighborhood method of point cloud and maintained a reasonable point density in network learning. Weinmann et al. [37] defined the neighborhood of point cloud in advance, which is independent of network training. By contrast, the purpose of this paper is to select neighborhood points while training the network.

**Figure 1:**
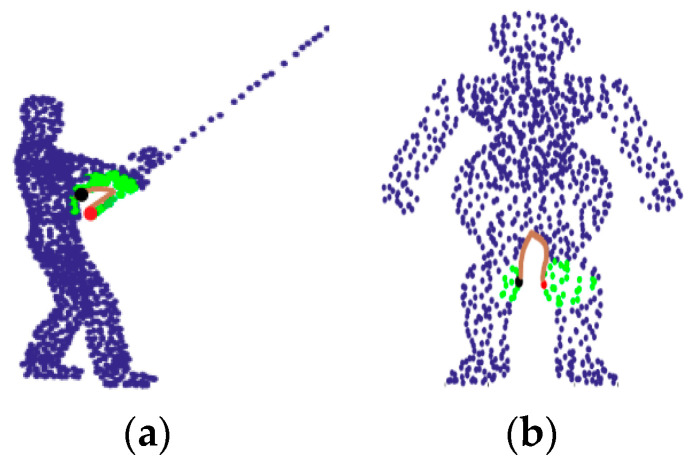
Examples of pathological neighborhood. (**a**) The pathological neighborhood of the fishing rod. (**b**) The pathological neighborhood of the knee.

The non-adaptive neighborhood selection, such as the *k*-NN method and spherical neighborhood method, may result in pathological neighborhoods. Figure 1 shows two point cloud models with such pathological neighborhoods, where the *k*-NN method is used to find the neighborhood (marked as green points) of the red point, and the brown line indicates the geodesic distance from the red point to one of its pathological neighborhood points (the black point). For the red point at fishing rod in Figure 1a, its correct neighborhood points should also be points at the fishing rod, but not the points representing the fisherman. For the red point on a man’s right knee in Figure 1b, the correct neighborhood points should be the points on the right knee, not the points on the left knee. Obviously, such pathological neighborhoods will lead to the network learning incorrect local information and further lead to pathological inferences. It is clear that discarding the pseudo neighborhood points with small Euclidean distance but large geodesic distance is helpful for the network to better understand the local surface information. Since surface-based geodesic topology is conducive to semantic analysis and geometric modeling of objects, He et al. [38] proposed deep geodesic networks for point cloud analysis.

Attention mechanism was used for weighting aggregation of point features in local regions [17,39,40,41], and it is also important for neighborhood learning. Chen et al. [17] used graph attention mechanism to learn local geometric representations of point clouds. Xie et al. [39] designed a self-attention module, which can realize the functions of feature transformation and feature aggregation. Feng et al. [40] proposed a Local Attention-Edge Convolution (LAE-Conv) to construct a local graph based on the neighborhood points searched in multi-directions. Xie et al. [41] used the local graph structure and the global graph structure to enhance the feature learning of point clouds. However, the traditional attention mechanism mainly focuses on using different features to obtain the weights of the neighborhood points, even for the pathological neighborhood as shown in Figure 1, such a kind of attention network also counts these pathological neighborhood points. By contrast, in this paper, the proposed attention mechanism will be used to evaluate the contribution degree of the neighborhood points, so as to filter out pseudo neighborhood points according to the evaluated contribution degree. Thus, it is necessary to consider which kind of features can be used to effectively obtain the contribution degree.

**Figure 2:**
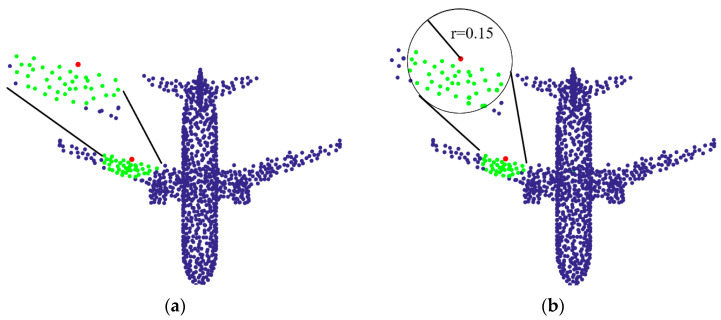
Neighborhoods obtained with two commonly used methods. (**a**) The *k*-NN neighborhood. (**b**) The spherical neighborhood with the radius *r* of 0.15.

Figure 2 shows the neighborhoods obtained with two common methods, in which the green points are the neighborhood points of the red point. The two methods are the *k*-NN neighborhood, and the spherical neighborhood, respectively. As shown in Figure 2, for the red point at the wing of the aircraft, theoretically, the network is expected to learn the features of the edge of the aircraft wing, rather than the features of the plane of this region. Therefore, it is better to remove points on the plane of the wing as much as possible to reduce the impact of these points on the network, but retain points at the edge of the wing. This indicates that the following problems are worth to be considered:

(1) How to choose the number of points in a neighborhood, and whether the number of neighborhood points of all points in a point cloud should be equal.
(2) If the neighborhood is determined, whether all points in the neighborhood help to understand the point cloud.
(3) Do these neighborhood points contribute equally to the correct understanding of point clouds?

Considering the pathological neighborhood in Figure 1 and unreasonable neighborhood in Figure 2, the motivation of this paper starts from the following two points:

(1) When the point cloud has pathological neighborhood (as shown in Figure 1), the network is expected to have the ability of learning the correct neighborhood points and discarding the pseudo neighborhood point.
(2) When the center point is at the edge (as shown in the red point in Figure 2), the network is hoped to learn the edge features of the point cloud instead of the plane features.

## 3. The Proposed Network

**Figure 3:**
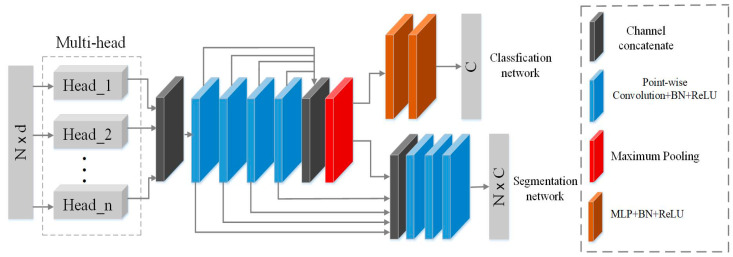
The architecture of the proposed Dynamic neighborhood Network (DNet).

Based on the above analyses, this paper propose a Dynamic neighborhood Network, denoted as DNet, to enhance neighborhood features learning for point cloud, so as to improve classification and segmentation of point cloud. Figure 3 shows the architecture of the DNet proposed in this paper, which has two branches: the classification sub-network and the segmentation sub-network. The core of the proposed DNet is a multi-head structure and its internal masking mechanism. Each head uses the attention mechanism to learn the contribution degree of each neighborhood point, and uses the masking mechanism to remove the neighborhood points with low contribution degree. Then, the weighted summation of the remaining neighborhood points is calculated to replace the maximum pooling of the neighborhood, so that the designed network has the ability to dynamically learn the effective neighborhood features of each point in the point cloud. Finally, multi-head structure composed of multiple single-head structures is used to learn multiple effective neighborhood features which are stacked as the final feature for subsequent point cloud classification and segmentation tasks.

Here, the neighborhood convolution of point cloud is first defined. Then, the multi-head structure in the proposed DNet is designed and its internal masking mechanism is described. Finally, the working principle and loss function of DNet are described.

### 3.1. Neighborhood Convolution

Given an unordered point set ***P*** in 3D space as a point cloud, where ***P*** = {*P_i_* | *i* = 1, …, *n*}, *P_i_* ∈ *R^d^* (generally, *d* = 3), which is the coordinate of the *i-*th point, denoted as *P_i_* = {*x*, *y*, *z*}, and *n* is the number of points in the point cloud. Then, let *N_all_*(*P_i_*) denote the neighborhood of the point *P_i_*,$N_{all}(P_{i})=\{P_{i}^{j}|j=1,\cdots ,k\}$, where $P_{i}^{j}$ is the *j*-th neighborhood point of *P_i_*, and *k* is the number of neighborhood points of the point *P_i_*. Since it is easy for the *k*-NN method to quickly construct a neighborhood graph, the *k*-NN neighborhood is used as the initial neighborhood in the proposed DNet. For the constructed neighborhood graph of *P_i_*, neighborhood learning can be performed on all points of *N_all_*(*P_i_*) to obtain the feature *F_all_*(*P_i_*) with respect to the point *P_i_* as follows

(1) $$F_{all}(P_{i})=Max(\sigma (h_{\theta }(P_{i}^{j}))),\;\forall P_{i}^{j}\in N_{all}(P_{i})$$

where *Max*(∙) is the max-pooling operation, σ(∙) is the activation function, and *h_θ_*(∙) is point-wise convolution with a set of learnable parameters *θ*. For 2D image, *h_θ_*(∙) can be a convolution kernel with the size of 3 × 3 and 5 × 5. However, for point cloud, since it is unstructured, *h_θ_*(∙) is a convolution kernel with the size of 1 × 1, which is called as point-wise convolution [20].

In order to make Equation (1) more generalized, it is modified as follows

(2) $$F_{all}(P_{i})=A(\sigma (h_{\theta }(P_{i}^{j},Oth))),\;\forall P_{i}^{j}\in N_{all}(P_{i})$$

where *A*(∙) is the aggregation function (such as the max-pooling, summing, averaging, etc.). "*Oth*" represents some additional information such as the density of the local region, the 3D Euclidean distance from the neighborhood point to the center point *P_i_*, etc. [35].

The traditional network only conducts neighborhood learning from all points of *N_all_*(*P_i_*) in the local region, no matter whether the points in the neighborhood are suitable or not. Therefore, this work tries to remove some of the points in the neighborhood *N_all_*(*P_i_*) through network learning, so as to adaptively obtain an effective neighborhood of the point *P_i_*, namely $N_{eff}(P_{i})=\{P_{i}^{j}|j=1,\cdots ,m\}$, *m* ≤ *k*. Thus, the more effective feature *F_eff_*(*P_i_*) of the point *P_i_* can be learned as follows

(3) $$F_{eff}(P_{i})=A(\sigma (h_{\theta }(P_{i}^{j})),\;\forall P_{i}^{j}\in N_{eff}(P_{i})\;\mathrm{and}\;N_{eff}(P_{i})\subseteq N_{all}(P_{i})$$

**Figure 4:**
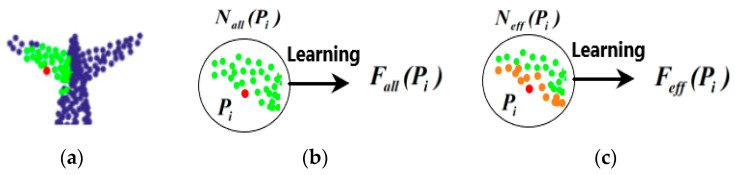
The diagram of the neighborhood learning. (**a**) Aircraft tail. (**b**) Feature learning on the *k*-NN neighborhood of the point *P_i_*. (**c**) Feature learning on the effective neighborhood (the orange points) which is more appropriate for feature learning of the edge of the wing.

As an example, Figure 4 shows the feature learning with two different neighborhood methods, where the green and orange points mark the neighborhood of the red point. In the figure, since the red point is located at the edge of the airplane wing, the feature of the red point should reflect the characteristics of the wing edge. It is seen that for the *N_all_*(*P_i_*), which is selected with *k*-NN method, some of the neighborhood points are not suitable for the feature learning of the wing edge. By contrast, the effective neighborhood *N_eff_*(*P_i_*) marked as the orange is more helpful for learning the features of wing edge. In other words, *N_eff_*(*P_i_*) is more expected for feature learning of the edge of the airplane wing compared with *N_all_*(*P_i_*).

### 3.2. Multi-Head Structure

The proposed DNet utilizes the attention mechanism and masking mechanism to learn the more effective feature *F_eff_*(*P_i_*). The main modules in the proposed DNet are the multi-head structure, which allows the network to learn information of different neighborhood ranges of the point clouds, that is, multi-scale features, so as to obtain sufficient context information and stabilize the network. Given a point cloud ***P***, the effective feature *F*(***P***) of the point cloud learned by the multi-head structure can be expressed as follows

(4) $$F(P)=\overset{m}{\underset{t=1}{\left|\right|}}F_{eff}{\left(P\right)}^{\left(t\right)}$$

where || is the multi-channel cascade operation, *m* is the number of heads, and *m* = 3 in this paper, *F_eff_*(***P***)^(*t*)^ denotes the effective feature learned by the *t-*th head. 

The proposed multi-head structure does not need to manually set multi-scale receptive fields as in [21]. For each head, as long as the number of initial neighborhood points in a neighborhood is set, an adaptive masking mechanism inside the heads will spontaneously filter out the neighborhood points with low contribution to obtain the features of different neighborhood ranges.

**Figure 5:**
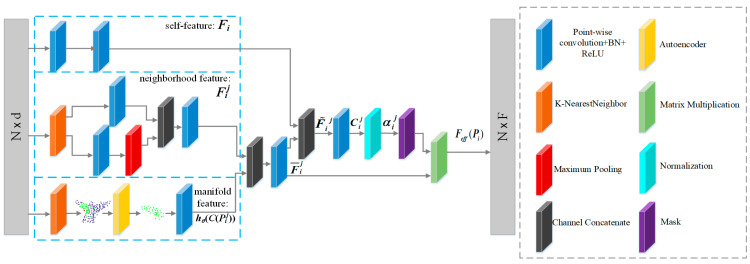
Single-head structure.

After designing the structure that captures multi-scale features, the next task is how to design the structure of each head so that it can select effective points in the neighborhood to promote network understanding of point cloud. Figure 5 shows the designed single-head structure. The attention mechanism can be used to obtain the feature of a point by weighted aggregation of features of the point’s neighborhood points. The attention mechanism will be used to assign a contribution degree to each point in the neighborhood, which indicates the contribution of the point to the learning of this local region. Therefore, the contribution of the neighborhood points can be identified according to the attention mechanism, based on which an adaptive masking mechanism can be designed. For a point *P_i_* ∈ ***P*** with the neighborhood *N_all_*(*P_i_*), the effective feature *F_eff_*(*P_i_*) of the point *P_i_* can be defined as follows

(5) $$F_{eff}(P_{i})=\sum_{j=1}^{k}M_{i}^{j}\cdot \alpha_{i}^{j}\cdot {\bar{F}}_{i}^{j}+b_{i}$$

where *α_i_^j^* is the contribution degree of the neighborhood point learned by the network, *b_i_* is the bias term, and *M_i_^j^* denotes an adaptive mask determined by the contribution degrees of the neighborhood. ${\bar{F}}_{i}^{j}$ is the integration feature that needs to be multiplied with the mask, it is composed of neighborhood features and manifold features, and defined as follows

(6) $${\bar{F}}_{i}^{j}=h_{\theta }(F_{i}^{j}⊕h_{\theta }(C(P_{i}^{j})))$$

where ⊕ represents channel concatenate, *C*(*P_i_^j^*) is the coding feature of *P_i_^j^*, and *h_θ_*(*C*(*P_i_^j^*)) is the manifold features of *P_i_^j^*. *h_θ_*(*C*(*P_i_^j^*)) is extracted from FELayer, which contains an autoencoding and point-wise convolution.

In order to establish the connection between different local regions, the covariance feature of the local region is added for each point *P_i_^j^* in the local region, and *F_i_^j^* can be expressed as follows

(7) $$F_{i}^{j}=h_{\theta }(COV(N_{all}(P_{i}))⊕P_{i}^{j})$$

In probability theory, covariance is used to measure the error between different variables, because it can well represent the statistical characteristics of the local regions. Therefore, the 3 × 3 covariance matrix of each region is calculated, and flattened to get a 9-dimensional covariance feature *COV*(*N_all_*(*P_i_*)), then it is concatenate with each point in the neighborhood to obtain the 12-dimensional data, which extends the neighborhood features of the point cloud.

The contribution degree *α_i_^j^* of the point *P_i_* is obtained through the feature ${\tilde{F}}_{i}^{j}$. ${\tilde{F}}_{i}^{j}$ learned inside each head is composed of two parts: the self-features $F_{i}$ and integration feature ${\bar{F}}_{i}^{j}$. Therefore, ${\tilde{F}}_{i}^{j}$ can be denoted as follows

(8) $${\tilde{F}}_{i}^{j}=F_{i}⊕{\bar{F}}_{i}^{j}$$

Then, for the point *P_i_* and its neighborhood point *P_i_^j^*, the weight *C_i_^j^* of the neighborhood point *P_i_^j^* is learned through the single-head structure as follows

(9) $$C_{i}^{j}=h_{\theta }({\tilde{F}}_{i}^{j})$$

Finally, in order to better compare the attention coefficients *C_i_^j^*, it is normalized as the contribution degree of the neighborhood points, which is defined as follows

(10) $$\alpha_{i}^{j}=\frac{\exp(C_{i}^{j})}{\sum_{l=1}^{k}\exp(C_{i}^{l})}$$

where *exp*(∙) is an exponential function, and *k* is the number of neighborhood points.

**Figure 6:**
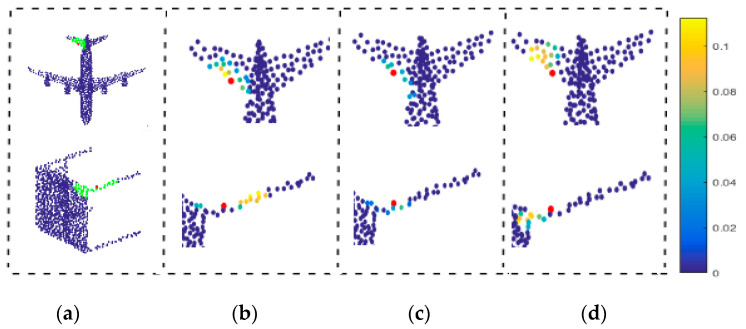
The contribution degree output from the three heads when the center point is an edge point. (**a**) Two point cloud models. (**b**) The first head. (**c**) The second head. (**d**) The third head.

In order to better understand the multi-head structure, Figure 6 shows the contribution degree of neighborhood points when the center point (red point) is an edge point. The contribution degree indicates how much the network learns from the neighborhood points of the red point. 

In Figure 6, as shown in the right colored bar, the closer the color of a neighborhood point is to yellow, the more features the network learns from the neighborhood point when processing the local region of the red point. Figure 6a shows the input models in which the green points indicate the initial neighborhood of the red point. Figure 6b–d show the contribution of the neighborhood points learned by the three heads to the center point. It is clear that the neighborhood range learned by each head is different. From the Figure 6, there are two points worth noting. Firstly, it is not that the closer the neighborhood point is to the red point, the more important it is; secondly, since the red point is at the edge of the airplane wing, the contribution degree of other edge points is significantly higher than that of the point on the wing plane. This indicates that the network is more willing to learn local features that are conducive to understand point clouds.

### 3.3. Masking Mechanism

As an important part of the multi-head structure, the masking mechanism is adopted to filter out the pseudo neighborhood points in the initial neighborhood so that the proposed network can learn neighborhood features more effectively. The adaptive mask *M_i_^j^* in Equation (5) can be expressed as follows

(11) $$M_{i}^{j}=\{\begin{array}{cc} 0, & if\;\alpha_{i}^{j}<T\\ \alpha_{i}^{j}, & otherwise \end{array}$$

where *T* is a threshold of the mask. The threshold can be obtained by different methods (e.g., the mean value of the weight of neighborhood points). If the contribution degree of a neighborhood point is less than the threshold, the point is regarded as the pseudo neighborhood point and will be removed from the neighborhood; otherwise, the neighborhood point is retained.

Assume that the dimension of the input point cloud is (*n*, 3), where *n* is the number of points with 3D coordinate (*x*, *y*, *z*). Ideally, the network is expected to be able to select *k_i_* neighborhood points of *P_i_* for effective neighborhood learning, and *k_i_* is different for the different center point *P_i_*. However, because the shape of the convolution kernel is fixed, the network cannot handle irregular data. For example, if the first point has 10 neighborhood points with the shape of (1,10,3), while the second point has 20 neighborhood points with the shape of (1,20,3), the network cannot stack these two points for learning. However, if both of the shapes of the two points is (1,20,3), the network can stack the two points into the shape of (2,20,3). Therefore, in this paper, the number of initial neighborhood points is fixed to *k*, and the mask *M_i_^j^* is used to remove the pseudo neighborhood points from the neighborhood since these points are not conducive to the network learning of the local region.

**Figure 7:**
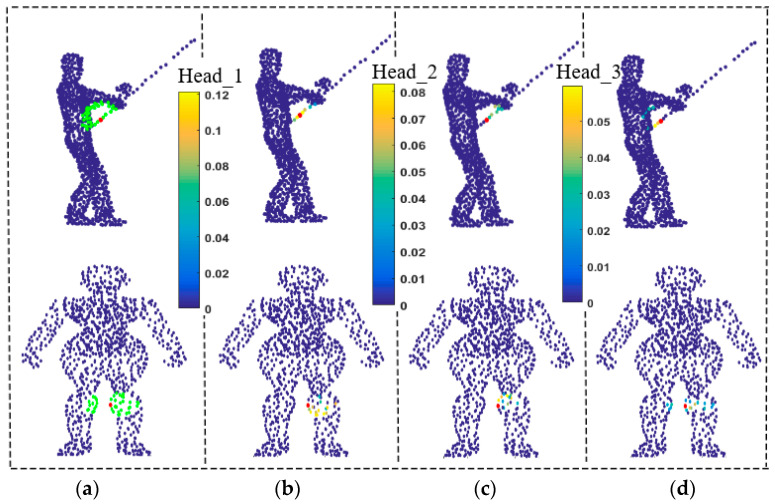
Neighborhood learning of multi-head structure under pathological conditions. (**a**) Two point cloud models. (**b**) The first head. (**c**) The second head. (**d**) The third head.

The traditional neighborhood learning methods do not consider the geodesic information, which may result in pathological neighborhood, as shown in Figure 1. By contrast, GeoNet [38] learns the point cloud with geodesic information to avoid learning pathological neighborhood features. For the proposed DNet, it can use the mask *M_i_^j^* to remove the neighborhood points with low contribution so that more effective neighborhood features can be learned even if only coordinate information of point cloud is available. This can effectively prevent the network from learning pathological region features such as the body or another knee in Figure 7a, where the green points are the initial neighborhood points of the red point. Figure 7b–d show the neighborhood points selected by the first, second and third heads, respectively. It can be seen from Figure 7 that the masking mechanism shields many pseudo neighborhoods points with large geodesic distance, thereby it effectively summarizes the neighborhood. Instead, if the pseudo neighborhood is not shielded by mask, the point cloud learning network will learn the wrong neighborhood information, leading to a decrease in the accuracy of classification or segmentation.

### 3.4. Learning with DNet

The architecture of the proposed DNet in Figure 3 can be used for point cloud classification (the upper branch) or segmentation (the lower branch). The point cloud classification sub-network in Figure 3 takes the coordinates of the whole point cloud as the input of the network, and after extracting multi-scale effective neighborhood features, it aggregates the point features through the max-pooling to output the classification results. The point cloud segmentation sub-network in Figure 3 concatenates global features with shallow features and outputs the segmentation results.

The core of the network consists of three heads, each of which can learn local information of different neighborhood ranges. Inside each head, the original local 3D space coordinates are used as the input, and the effective neighborhood features are learned as the output. The head obtains the attention weight of the neighborhood points by learning self-features, manifold features and neighborhood features. Then, the mask is used to remove some pseudo neighborhood points to obtain dynamic neighborhood features. Finally, a multi-head structure is used to learn multiple effective neighborhood features and stack them as the final feature for subsequent classification and segmentation tasks.

### 3.5. Loss Function

In this paper, an autoencoder is used to extract the 2D manifold features of the point clouds. Usually, for reconstruction networks whose purpose is to reconstruct the entire point cloud model, the complex loss of Chamfer Distance (CD) or Earth Mover’s distance (EMD) are used as the loss function because of the disorder of point cloud. However, the task of this paper is not to reconstruct the entire point cloud model, but to roughly reconstruct the shape of the local neighborhood so as to extract the 2D manifold features of the point clouds. Therefore, since the local neighborhood is generally with simple topological structure, a simple L2 loss function is used in this work, and expressed as follows

(12) $$L_{AE}=\sum_{i=1}^{n}\sum_{j=1}^{k}{\left(P_{i}^{j}-{\tilde{P}}_{i}^{j}\right)}^{2}$$

where ${\tilde{P}}_{i}^{j}$ is the reconstructed point of $P_{i}^{j}$.

**Figure 8:**
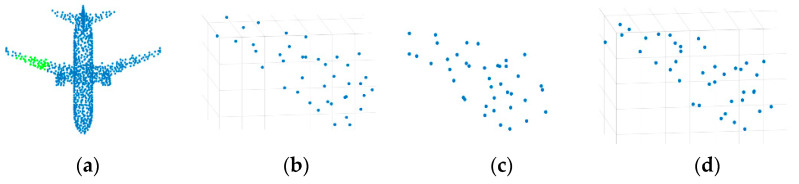
Compression and reconstruction of point cloud by using autoencoder with L2 loss function. (**a**) 3D point cloud. (**b**) 3D points at the green area of (**a**). (**c**) Compressed result of (**b**). (**d**) 3D points reconstructed from (**c**).

Figure 8 illustrates the effectiveness of the autoencoder with L2 loss function. We draw a grid in the figure to distinguish 3D points from 2D points. Figure 8a is the original input 3D point cloud, and Figure 8b enlarges the green local neighborhood in Figure 8a. Figure 8c depicts the result of using an autoencoder to compress Figure 8b to a 2D plane, and Figure 8d depicts the 3D points reconstructed from Figure 8c. It is clear that even though the simple L2 loss function is used instead of the more complex loss function in the autoencoder, the shape of the reconstructed 3D points is similar with that of the original shape of the local neighborhood.

Let *y* be the label of point cloud classification or segmentation, and $\hat{y}$ be the prediction result of DNet. The loss function of point cloud classification or segmentation is $L_{task}=-y\cdot \log(\hat{y})$, the final loss function of the proposed DNet is defined by

(13) $$L_{total}=L_{task}+L_{AE}$$

## 4. Experimental Results and Discussions

In this section, the training configuration of the networks is first introduced, and then the proposed DNet is tested on the benchmark dataset ModelNet40 [42] for point cloud classification, and on the benchmark datasets ShapeNet [43] and S3DIS [44] for point cloud segmentation, compared with other deep learning networks.

### 4.1. Network Training

The proposed DNet is constructed on Tensorflow, and the experiments are implemented on a computer with Intel Core I7-7820X CPU (3.6 GHz, 128GB memory) and GeForce RTX2080Ti GPUs. For the point cloud classification, 1024 points are uniformly sampled from the 3D grid of each point cloud as the network input, and the number of initial neighborhood points, that is, *k,* is set to 40. For part segmentation and indoor segmentation of point cloud, the number of input points of the DNet is 2048 and 4096, respectively, and *k* is set to 50. For the multi-head structure, in total three heads are used, and the output dimension of each head is 16. During the training phase, Adaptive Moment Estimation (ADAM) solver is used with the base learning rate of 0.001, the learning rate decay is executed every 40 epochs. ReLU and batch normalization are applied after each layer except the last fully connected layer. For the classification dataset, 200 epochs are trained with the batchsize of 32; while for the segmentation datasets, 100 epochs are trained with the batchsize of 16.

### 4.2. Point Cloud Classification

The performance of the proposed DNet on point cloud classification is tested on the ModelNet40 dataset [42]. This dataset contains 40 categories, including beds, chairs, airplanes, etc., with a total of 12,311 3D mesh models. In the experiments, 9843 models in the ModelNet40 dataset are used as the training set, while the remaining 2468 models constitute the testing set. For each model, 1024 points are uniformly sampled from the grid model and normalized into the unit circle. During the training, data augmentation techniques are used to scale point clouds in the range of [0.8, 1.25] and translate the point clouds in the range of [−0.1, 0.1].

Table 1 shows the classification results of the proposed DNet compared with the other sixteen advanced networks. As shown in the “input” column of Table 1, the methods, including the Spec-GCN [15], Pointconv [6], AGCN [41], PointNet++ [21], SpiderCNN [7] and SO-Net [28], require coordinates of point cloud as well as normal information as the input of their networks, while the other eleven comparison networks and the proposed DNet only need the coordinates of point cloud. Moreover, the networks listed in the last three (PointNet++ [21], SpiderCNN [7] and SO-Net [28]) for comparison use 5k points, rather than 1k points as other networks do. To evaluate the performance of different networks, the mean accuracy of each class of point cloud classification (mA) and the overall accuracy of point cloud classification (OA) are used, as shown in Table 1. It can be found that the proposed DNet has achieved good results. However, for most of the networks in Table 1, their focus is not on the effective neighborhood selection, which is emphasized by the proposed DNet. Therefore, in order to make a fairer comparison, the proposed DNet is mainly compared with DGCNN [35] and the PointNet++ [21] without normal information, because DGCNN also utilizes the *k*-NN neighborhood while PointNet++ adopts a spherical neighborhood. Table 1 shows that in terms of OA, the proposed DNet has 1.4% and 2.9% improvement over the DGCNN and the PointNet++ without normal information, respectively. It illustrates the importance of effective neighborhood selection for feature learning in the learning-based point cloud classification methods.

**Figure 9:**
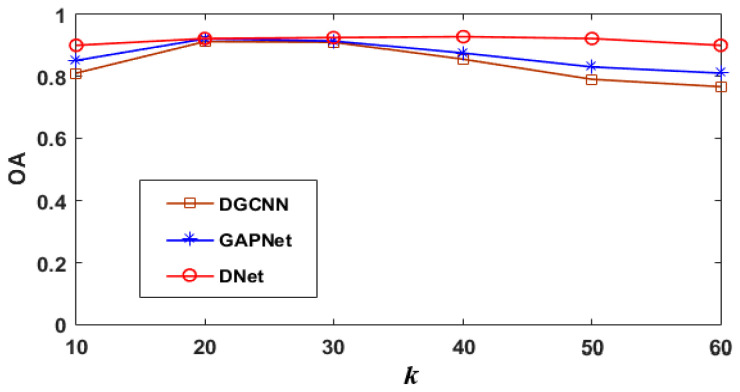
The influence of the number of initial neighborhood points on classification accuracy.

To test the influence of the number of initial neighborhood points *k* on the networks, GAPNet [17], DGCNN [35], and the proposed DNet are compared with each other, and all of them are the *k*-NN neighborhood-based networks. In the experiments, *k* is set to 10, 20, 30, 40, 50, and 60, respectively, and the networks are trained at each *k* separately, without using any data augmentation techniques. Figure 9 gives the corresponding OAs of the three networks with respect to each *k*. As shown in Figure 9, GAPNet and DGCNN achieve their highest accuracy when *k* is 20, and then the accuracy decreases with the increase in *k*. By contrast, the proposed DNet can achieve higher accuracy under more neighborhood points benefiting from the attention mechanism and masking mechanisms, and the highest accuracy is achieved when *k* is 40. On one hand, more initial neighborhood points can ensure that there are enough points describing the local region to be included in the network learning. On the other hand, the masking mechanism can filter out the pseudo neighborhood points with low contribution which are not conducive to the correct learning of the network. Therefore, the proposed DNet achieves higher classification accuracy.

**Figure 10:**
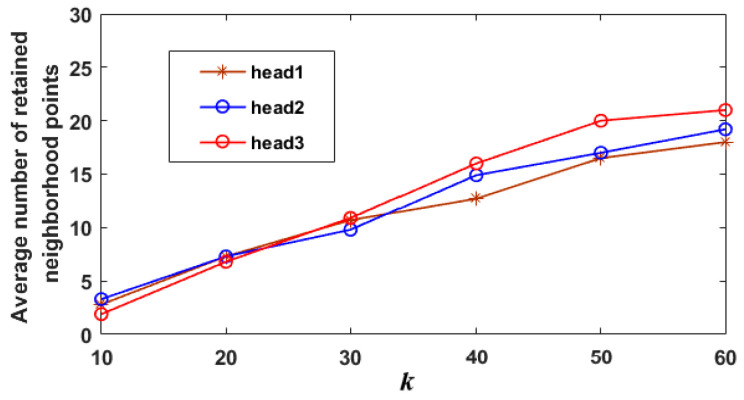
The average number of neighborhood points retained by the multi-head structure.

Additionally, in order to further analyze the influence of the number of initial neighborhood points on the performance of multi-head structure, the average numbers of the neighborhood points retained by the three heads of DNet are calculated, as shown in Figure 10, where all “airplane” models are used for the calculation. It should be noted that the neighborhood points retained by the three heads are the real learning content of the network. As shown in Figure 10, when the number of initial neighborhood points, *k*, is small, the average numbers of neighborhood points retained by the three heads are similar, and this will reduce the ability of the multi-head structure to capture multi-scale features. However, when *k* reaches 40, 50 or 60, the difference of the number among the three heads is obvious, indicating that the multi-head structure can capture multi-scale features. However, if *k* is too large, it will increase the burden of searching neighborhood and wash out high-frequency features [45], so *k* is set to 40 in this work. 

In the proposed DNet, the multi-head structure is utilized to learn multi-scale neighborhood features. However, too many heads will increase the complexity of the network. Therefore, to balance the complexity and accuracy, the number of head *N* is set to 3 in this paper. We have also tested the computational complexity of the proposed DNet with *N* = 3, compared with PointNet [20], PointNet++ [21] and DGCNN [35]. The comparison experimental results are given in Table 2. PointNet is not a neighborhood-based method, and it has the lowest complexity but also lowest classification accuracy in Table 2. PointNet++ and DGCNN are the representations of spherical neighborhood and *k*-NN neighborhood-based methods, respectively. In this experiment, for DGCNN, the number of neighborhood points *k* is 20, which is the default set by the author, while for the proposed DNet, *k* is set to 40. For PointNet++, the default parameters are used. It is seen that compared with the other networks, the proposed DNet is more lightweight, faster and more accurate.

As a very important part of DNet, the masking mechanism can remove the pseudo neighborhood points in the initial neighborhood to achieve effective feature learning. There are some different kinds of masking mechanisms: for example, the mean masking and median masking mechanisms. The mean masking mechanism uses the average of contribution degrees of all the initial neighborhood points as the threshold to remove the pseudo neighborhood points. However, in the median masking mechanism, the median is used as the threshold instead of the average, and therefore the number of retained neighborhood points is fixed. Table 3 gives the point cloud classification results with respect to the two different masking mechanisms. The median masking mechanism is superior to the no masking scheme but inferior to the mean masking mechanism because of the fixed number of retained neighborhood points. Therefore, the mean masking mechanism is used in this paper. The experimental results indicate that not all points in a local region are helpful to network learning, in fact, some of them may weaken the learning and understanding ability of the network to point cloud processing.

### 4.3. Point Cloud Segmentation

Point cloud segmentation is a fine-grained recognition task that requires understanding the role of each point playing in its respective category, so it is one of the challenging point cloud processing tasks.

#### 4.3.1. Part Segmentation of Point Cloud

The part segmentation is tested on a ShapeNet dataset [43], which has 16,881 models in 16 categories, with 50 annotated parts in total. In the experiments, for each model in the ShapeNet dataset, 2048 points are extracted as the input of the networks. On the premise that the model category is known, the one-hot encoding of the category is concatenated to the last feature layer as the input of the fully connected layer in DNet, and finally the prediction result is obtained.

**Figure 11:**
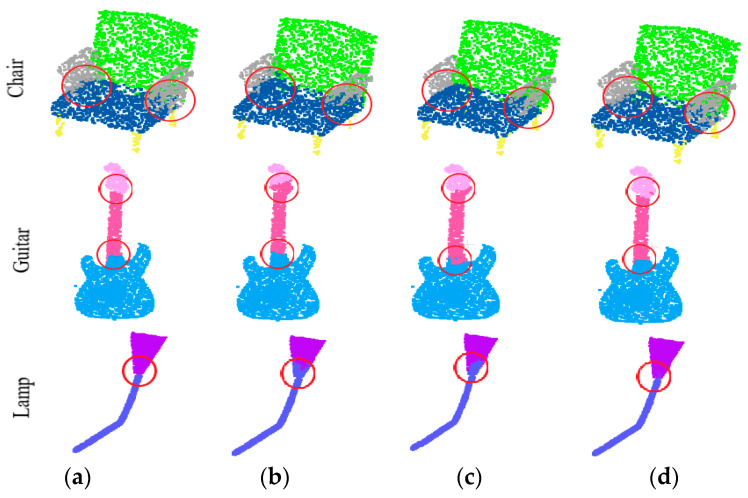
Part segmentation results of three models in ShapeNet dataset. (**a**) Ground Truth. (**b**)PointNet++. (**c**) Dynamic Graph Convolutional Neural Network (DGCNN). (**d**) the proposed DNet.

Intersection over Union (IoU) is used to evaluate the performance of the proposed DNet and other comparison networks. The IoU of a class refers to the average of all IoUs with respect to such kind of objects, denoted as class mean IoU (cIoU). The average of cIoU of all classes is denoted as mcIoU. The average IoU of all classes refers to the average of the IoU of all test objects, denoted as instance mean IoU (mIoU). Table 4 gives the cIoU, mcIoU and mIoU results of several different networks implemented on ShapeNet dataset, and the best results are shown in bold. Compared to the PointCNN [25] which is not a neighborhood-based method, the proposed DNet has demonstrated its potential, surpassing in several categories. For the sake of fairness, the proposed DNet is further compared in detail with the two representative neighborhood-based learning networks, that is, PointNet++ and DGCNN. PointNet++ does not consider how to learn effective regional features, but simply stacks features in multiple ranges; its mcIoU and mIoU are of 81.9% and 85.1%, respectively. Although DGCNN considers the neighborhood information of both the spatial and feature spaces, it does not consider which features of the neighborhood points are effective, its performance of mcIoU and mIoU is 82.3% and 85.2%, respectively. By contrast, the proposed DNet can reasonably learn the effective neighborhood information to achieve better results. We also carried out a qualitative analysis, and the visualization results of the components were visualized in Figure 11.

Figure 11 shows some of the part segmentation results, where Figure 11a shows the ground truth of the part segmentation. In Figure 11, the parts marked with red circles are segmented incorrectly by PointNet++ and DGCNN, while the segmentation results achieved by the proposed DNet are consistent with the ground truth. The segmentation results of PointNet++ and DGCNN at some of the parts of the connection are incorrect, while the DNet can predict these parts better. From the perspective of an effective neighborhood, the proposed DNet assigns lower contribution degree to the neighborhood point whose label is different from that of the central point, thereby the segmentation accuracy of the proposed DNet is improved.

#### 4.3.2. Scene Segmentation of Point Cloud

For scene segmentation, comparative experiments are implemented on S3DIS dataset [44]. The dataset has six areas, including 271 indoor scenes (for example conference room, hallway, office etc.) with a total of 13 types of objects (such as chair, table, floor, wall and so on). In S3DIS dataset, each point has nine attributes: XYZ space coordinates, RGB color information, and a normalized location in the room. In the experiments, the same training strategy as in PointNet [20] is adopted, and 4096 points are randomly sampled from the scene as the network input.

**Figure 12:**
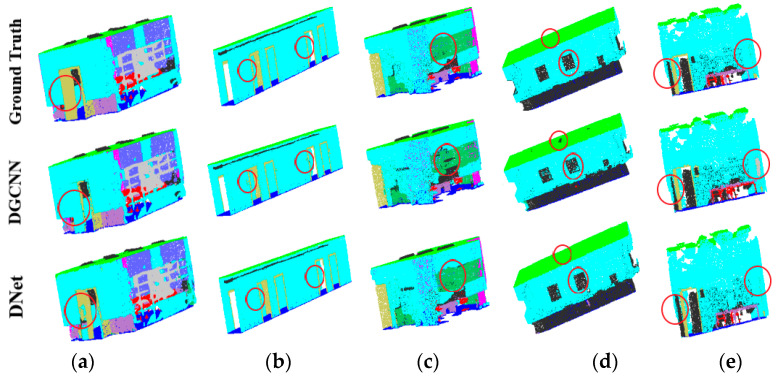
Comparison of point cloud segmentation with indoor scenes in the S3DIS dataset. (**a**) ConferenceRoom. (**b**) Hallway. (**c**) Office. (**d**) Pantry. (**e**) Storage.

In the experiments, 6-fold cross validation is adopted to verify the performance of the comparison networks. In this case, five areas of S3DIS dataset are used for training while the remaining one area is for testing. Then, the average results of the six tests are reported as the indicators of the performance of the networks, as shown in Table 5. In this table, the experimental results of the comparison networks also come from the corresponding literature. Considering that some of the networks only provided the experimental results of the segmentation of Area 5, that is, only Area 5 is used for testing while the other five areas are used for training, we also show such experimental results in Table 6. In Table 5 and Table 6, the best results are in bold. It is seen that the proposed DNet achieves better results compared with other networks except the PointCNN and PCCN. Figure 12 shows the scene segmentation results obtained with different learning networks. It is seen that for the points in red circles, the segmentation achieved by the proposed DNet is closer to the label compared with the DGCNN.

PointCNN transforms the point cloud into the feature space by learning an X-matrix, and then weights and sums it using traditional convolution. This method maintains the invariance of the displacement of the point cloud in the feature space. When the point cloud is rotated or translated, PointCNN can still capture the fine-grained information of each point, so it achieves better results in point cloud segmentation. By contrast, the proposed DNet learns the point cloud from the perspective of the neighborhood and also shows its competitive performance. Compared with PointNet++ and DGCNN, which are also neighborhood-based learning networks, DNet achieves better performance in classification and segmentation of point cloud. This indicates that both of point cloud permutation invariance and effective neighborhood learning are indispensable for deep learning-based point cloud processing.

### 4.4. Ablation Experiments

**Figure 13:**
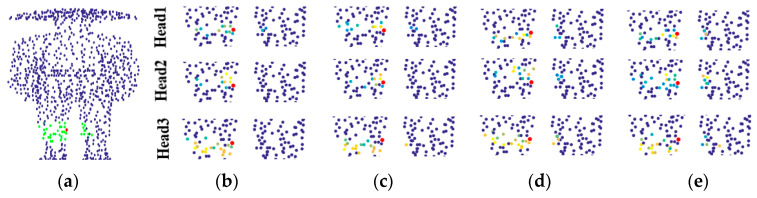
Neighborhood points selected by multi-head structure with different features. (**a**) Model. (**b**) All features. (**c**) Without self-features. (**d**) Without manifold features. (**e**) Without neighborhood features.

To clearly show the effect of the three different kinds of features in DNet, ablation experiments are implemented, and the results are given in Table 7. It is seen that if the neighborhood features are absent, the classification accuracy of DNet is significantly reduced, implying that the neighborhood features are very important for the network to understand the point cloud. Figure 13 gives the visualized results of the neighborhood points selected by the proposed DNet in the absence of some features. In Figure 13, the self-features have relatively less influence on neighborhood point selection, while neighborhood features, manifold features and neighborhood features can improve the performance of the DNet.

### 4.5. Robustness Analysis

**Figure 14:**
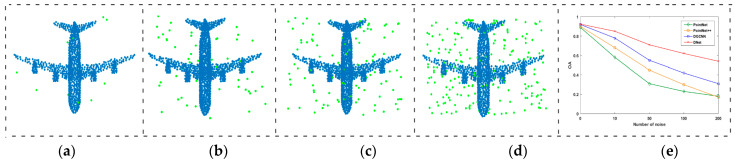
The influence of noise on the classification accuracy of different networks. (**a**) 10 noise points. (**b**) 50 noise points. (**c**) 100 noise points. (**d**) 200 noise points. (**e**) Classification accuracy.

In order to verify the robustness of the proposed DNet, uniform noise is added to the point cloud models in the testing set of the ModelNet40 dataset, and the number of noise points is set to 10, 50, 100 and 200, respectively, as shown in Figure 14a–d. Since the input points of networks are uniformly sampled from the point cloud model and normalized into the unit circle, the coordinates of the added noise points are also limited to the range of [−1, 1]. The training set is noise-free, and the data augmentation is not used in the training process. The final result is shown in Figure 14e, where the abscissa is the number of noise points, and the ordinate denotes the overall accuracy of classification of a network. For the four comparison networks, it is seen that the classification accuracy decreases at different rate with the increase in the number of noise points. PointNet does not consider the neighborhood, so it is most affected by noise points. PointNet++ and DGCNN are relatively better than PointNet. By contrast, the proposed DNet further considers the dynamic neighborhood, so it has strong robustness to noise compared with the other three networks.

## 5. Conclusions

In view of the lack of an effective learning network for point cloud neighborhood selection, a new Dynamic neighborhood Network, known as DNet, has been proposed to extract effective neighborhood features in this paper. The proposed DNet has a multi-head structure with two important modules: the Feature Enhancement Layer (FELayer) and the masking mechanism. The FELayer enhances the manifold features of the point cloud, while the masking mechanism can suppress the effects of some pseudo neighborhood points, so that the network can learn features that are conducive to understanding the local geometric information of the point cloud. In order to obtain sufficient contextual information in the proposed DNet, the multi-head structure is designed to allow the network to autonomously learn multi-scale features of a local region. The experimental results on three benchmark datasets have proved the effectiveness of the proposed DNet. The visualization results also show that the proposed DNet can capture more effective neighborhood features that are easy to understand.

## Figures and Tables

**Table 1:** Classification accuracy of different networks (%). (mA and OA denote the mean accuracy of each class of point cloud classification and the overall accuracy of point cloud classification, respectively.).

Method	Input	Points	mA	OA
Pointwise-CNN [23]	xyz	1k	81.4	86.1
ECC [18]	xyz	1k	-	87.4
PointNet [20]	xyz	1k	86.2	89.2
SCN [39]	xyz	1k	87.6	90.0
Kd-Net [14]	xyz	1k	86.3	90.6
PointNet++ [21]	xyz	1k	-	90.7
KCNet [34]	xyz	1k	-	91.0
Spec-GCN [15]	xyz	1k	-	91.5
PointCNN [25]	xyz	1k	88.1	92.2
DGCNN [35]	xyz	1k	90.2	92.2
GAPNet [17]	xyz	1k	89.7	92.4
Spec-GCN [15]	xyz+normal	1k	-	91.8
Pointconv [6] AGCN [41]	xyz+normal xyz+normal	1k 1k	- 90.7	92.5 92.6
PointNet++ [21]	xyz+normal	5k	-	91.9
SpiderCNN [7]	xyz+normal	5k	-	92.4
SO-Net [28]	xyz+normal	5k	90.8	93.4
DNet	xyz	1k	90.9	93.6

**Table 2:** Comparison of different methods on model complexity, forward time, and classification accuracy.

Method	Model Size (MB)	Time (ms)	Accuracy (%)
PointNet [20]	40	6.7	89.2
PointNet++ [21]	12	21.3	90.7
DGCNN [35]	21	24.6	92.2
Proposed DNet	17	19.2	93.6

**Table 3:** Effect of different masking mechanisms on point cloud classification (%).

Mask	mA	OA
No mask	92.9	89.2
Median mask	93.3	90.1
Mean mask	93.6	90.9

**Table 4:** Comparison of part segmentation of point cloud (%).

Method	mcIoU	mIoU	cIoU															
			Air Plane	Bag	Cap	Car	Chair	Ear Phone	Guitar	Knife	Lamp	Laptop	Motor Bike	Mug	Pistol	Rocket	Skate Ball	Table
Kd-Net [14]	77.4	82.3	80.1	74.6	74.3	70.3	88.6	73.5	90.2	87.2	81.0	94.9	57.4	86.7	78.1	51.8	69.9	80.3
PointNet [20]	80.4	83.7	83.4	78.7	82.5	74.9	89.6	73.0	91.5	85.9	80.8	95.3	65.2	93.0	81.2	57.9	72.8	80.6
SPLATNet [24]	82.0	84.6	81.9	83.9	88.6	79.5	90.1	73.5	91.3	84.7	84.5	96.3	69.7	95.0	81.7	59.2	70.4	81.3
KCNet [34]	82.2	84.7	82.8	81.5	86.4	77.6	90.3	76.8	91.0	87.2	84.5	95.5	69.2	94.4	81.6	60.1	75.2	81.3
GAPNet [17]	82.0	84.7	84.2	84.1	88.8	78.1	90.7	70.1	91.0	87.3	83.1	96.2	65.9	95.0	81.7	60.7	74.9	80.8
RSNet [26]	81.4	84.9	82.7	86.4	84.1	78.2	90.4	69.3	91.4	87.0	83.5	95.4	66.0	92.6	81.8	56.1	75.8	82.2
SpiderCNN [7]	82.4	85.3	83.5	81.0	87.2	77.5	90.7	76.8	91.1	87.3	83.3	95.8	70.2	93.5	82.7	59.7	75.8	82.8
AGCN [41]	82.6	85.4	83.3	79.3	87.5	78.5	90.7	76.5	91.7	87.8	84.7	95.7	72.4	93.2	84.0	63.7	76.4	82.5
SCN [39]	-	84.6	83.8	80.8	83.5	79.3	90.5	69.8	91.7	86.5	82.9	96.0	69.2	93.8	82.5	62.9	74.4	80.8
PointCNN [25]	84.6	86.1	84.1	86.5	86.0	80.8	90.6	79.7	92.3	88.4	85.3	96.1	77.2	95.3	84.2	64.2	80.0	83.0
PointNet++ [21]	81.9	85.1	82.4	79.0	87.7	77.3	90.8	71.8	91.0	85.9	83.7	95.3	71.6	94.1	81.3	58.7	76.4	82.6
DGCNN [35]	82.3	85.2	84.0	83.4	86.7	77.8	90.6	74.7	91.2	87.5	82.8	95.7	66.3	94.9	81.1	63.5	74.5	82.6
DNet	83.8	86.1	84.5	85.2	88.6	79.3	91.7	77.8	91.5	88.7	84.7	95.7	73.4	95.3	82.3	62.8	76.8	82.1

**Table 5:** Scene segmentation results on S3DIS dataset evaluated with 6-fold cross validation (%).

Method	OA	mA	mIOU
PointNet [20] SCN [39]	78.5 81.6	66.2 -	47.6 52.7
DGCNN [35]	84.1	-	56.1
RSNet [26]	-	66.4	56.4
AGCN [41] SPGraph [19]	84.1 85.5	- 73.0	56.6 62.1
PointCNN [25]	88.1	75.6	65.3
DNet	86.3	75.3	66.7

**Table 6:** Scene segmentation results of Area 5 in S3DIS dataset (%).

Method	OA	mA	mIOU
PointNet [20]	-	49.0	41.1
SegCloud [27]	-	57.4	48.9
PointCNN [25]	85.9	63.9	57.3
SPGraph [19]	86.4	66.5	58.0
PCCN [32]	-	67.0	58.3
DNet	86.5	66.3	59.7

**Table 7:** Classification accuracy of DNet using different features on ModelNet40 dataset.

DNet Using Different Features	OA
without self-features	93.0
without manifold features	92.3
without neighborhood features	90.2
all features	93.6

## Data Availability

Not applicable.
